# Model based development of temperature control schemes for fighter aircraft ECS

**DOI:** 10.1016/j.heliyon.2022.e11727

**Published:** 2022-11-21

**Authors:** Sathiyaseelan Arunachalam, Arul Mozhi Selvan Varadappan

**Affiliations:** a Aircraft Research and Design Centre, Hindustan Aeronautics Limited, Bangalore, India; b Advanced Automotive Research Laboratory, Department of Mechanical Engineering, National Institute of Technology, Tiruchirappalli, India

**Keywords:** Environmental control system, Fighter aircraft, PID, Simulation, Temperature control

## Abstract

The performance of Temperature Control System (TCS), a sub-system of an advanced fighter ECS (Environmental Control System) is studied for its operations both in on-ground and in-air conditions. The standard Bootstrap air cycle system is considered with ACM (Air Cycle Machine) as the main component, which consists of a compressor and a turbine, to produce the cold air. ECS, in particular TCS, is responsible in maintaining the required cockpit or cabin temperature. As the fighter aircraft doesn’t require cabin air recirculation, the cabin temperature depends solely on the supply air thermal conditions. The cabin temperature regulation is realized by controlling the supply conditions by maintaining required temperature at ACM outlet and at the entrance duct to the cabin. These temperatures are critical parameters that necessitates appropriate controller to keep the cabin in a good comfortable state. ECS of a fighter aircraft is highly complex and nonlinear than commercial aircraft. The control logics applied, on this system, will drive the actuators or TCVs (Temperature Control Valves) to keep the requisite cabin temperature. The control logics applied on the controller to be simple and appropriate to meet the requirements of fighter aircraft ECS. This shall minimize the control cycle oscillation and subsequent supply air temperature instabilities. This paper explains novel cabin temperature control schemes and their influence on the ECS performance especially during the transient operation. The complete architecture of the control schemes along with system components are modelled in AMESim and the comparison is made for different operating conditions. Finally, a novel variable time-delay based method to control the cabin supply air temperature is proposed.

## 1 Introduction

Aircraft system design has been improved recent years and advanced systems and their control technologies play key roles in futuristic aircraft development. Environmental Control System (ECS), which controls the comfort conditions inside the cabin, generally uses engine bleed air as its source [1]. While some progress happens to have bleed less or more electric ECS, majority uses the bleed air. While bleed air extracted from high pressure stage and an intermediate stage of the engine in modern aircraft, majority of fighter aircraft ECS extract air from single high pressure stage. The bleed temperature and pressure, at the tapping point, from the engine vary extensively due to change in engine operating conditions [2, 3]. The general bleed parameters are in the range of 260 kPa–1700 kPa & 473 K [4, 5] for commercial aircraft and exceed 2068 kPa (300 psi) & 873 K for combat aircraft [6, 7]. Due to the above, the bleed air system of fighter aircraft is complex than civil aircraft. Fighter aircraft executes complex flight profiles in extreme ambient conditions and seeks efficient and simple control system for the monitoring and control of its subsystems. Due to extreme operating conditions and to meet complex flight requirement, off-design characteristics also need to be considered for the design of subsystems and that too at the early stage of development [8].

Several literatures are published mainly on the commercial aircraft Environmental Control System to explain its performance characteristics based on simulation and/or experiments. Pérez-Grande et al. [9] presented on the geometry optimization of an ECS component to reduce the entropy generation and to reduce the system weight. The control valves, in their model, kept fully open for the entire analysis, which indicates that they are not part of the control logics. Rubens Romani et al. [10] modelled the ECS and simulated for constant bleed input and the system performance was presented for on-ground cooling and heating cases. WANG Jiali et al. [11] presented about controlling the ECS pack outlet temperature to meet the requirement for on-ground operation. The temperature fluctuation, for the supply air, is appeared to be more. Pollok et al. [12] related various bleed air temperature control strategies. They recommended MAP based method to control the bleed air temperature.

Alexander Pollok, in his other work [13, 14], used zone temperature control scheme and presented the results for PID with Control-Input Normalization (CIN). The results indicate a significant improvement in the performance. Jian et al. [15] presented fuzzy logic based control methods to control the cabin temperature. In their research, pull time of the control actuator relay is compensated and the control signal is derived from fuzzy logic table. This control signal is based on the temperature difference of the actual and the required, and its rate of change [26]. By way of this, the motor rotation angle is controlled by the relay and subsequently the cabin temperature. Zhongdi et al. [16] worked on the dynamic simulation of TCS of a commercial aircraft. In their work, the system was modelled in Matlab/Simulink and adopted PID control algorithm to control cockpit and cabin temperature zones.

All these presented works explain the TCS of commercial aircraft. Temperature control system of military aircraft not considered and not explained. Also, the impact of the control strategies on system performance and their implications not explained adequately. This paper explains a novel control strategy in the design of cabin TCS. A modern fighter aircraft’ cabin temperature control system, the sub system of ECS to control the cockpit supply air temperature, is studied for both on ground and in air operations [27, 28, 29]. Various control schemes on cabin temperature control are studied based on the software model presented in the work [17, 18]. Oscar Camacho and Hugo Leiva [19] presented about the time delays in the control system in two broad categories. The first kind of delay is from sensors or measurements which is called as delay in output or delay in state. The other one is from the control action which is called as delay in control or delay in input. They proved the approximate controllability of the semi linear heat equation. They used either delay in input or output with the help of Rothe’s theorem. In our work, the delay in control namely Time-delay (TD) control, as presented in the work [18] is compared with PID for various time-delays and using two different drive times for the control actuators. Based on the results of the above, an improved version of TD, i.e. Variable Time-delay has been introduced. In this variable TD control, the time-delay between successive control signals to the actuator is varied according to the bleed air input parameters. The bleed air pressure and its rate of change are considered to determine the value of time delay which is then used for the control of the actuators.

## 2 System description

Aircraft ECS maintains the required temperature and pressure of the occupied compartments and/or cockpit. It controls supply air temperature and the flow to meet these requirements. The system works on Bootstrapcycle by taking bleed air as the input. Unlike commercial aircraft, in this fighter, the bleed air is drawn from the engine compressor from its last stage and passes through heat exchangers, pipe lines, Air Cycle Machine (ACM) which consists of a turbine and a compressor, and control valves and then supplied into the cockpit [20, 25]. Initially the bleed air is cooled at PRC (Pre-cooler) then at PHE (Primary Heat Exchanger) and supplied to the ACM. To maintain the temperature limits at the downstream of PHE, a by-pass line connects directly to the compressor along with a by-pass valve.

**Figure 1:**
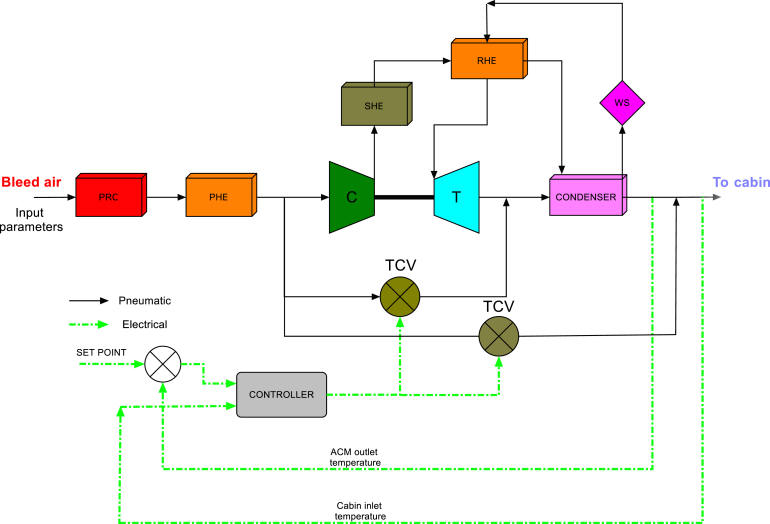
Schematic of cabin temperature control system.

After compression, the air passes through Secondary Heat Exchanger (SHE), reheater and the hot side of condenser. After this, the cooled humid air passes through the high pressure water separator (WS) and re-heater then enters the turbine. A portion of hot bleed air mixes with turbine outlet to meet the temperature requirement at the end of ACM and prevent ice formation [21]. This by-pass air flow is controlled with the help of a TCV (Temperature Control Valve). The turbine downstream or ACM outlet temperature, governs the TCV angle. The schematic of temperature control system for the cockpit is given in Figure 1. The TCV control signal is based on the error between measured ACM outlet temperature and the required temperature value. Based on the error, the controller calculates the required control signal to drive the TCV. The TCV is used at two places, one as a bypass valve to regulate the temperature at the downstream of the ACM and other as a bypass valve to maintain cabin inlet temperature. This by-pass line along with the TCV connects hot air stream at the ACM compressor inlet with condenser downstream to meet the requirement of cabin supply air temperature. Based on the temperature measurement at the condenser downstream after mixing, the controller computes the cabin mean temperature and accordingly controls this TCV. The cabin mean temperature is calculated as below in Eq. (1).

(1) $$T_{cab\;mean}=0.25\;T_{cabin\;inlet}+0.75\;T_{cabin\;outlet}$$

## 3 Cabin temperature control

**Figure 2:**
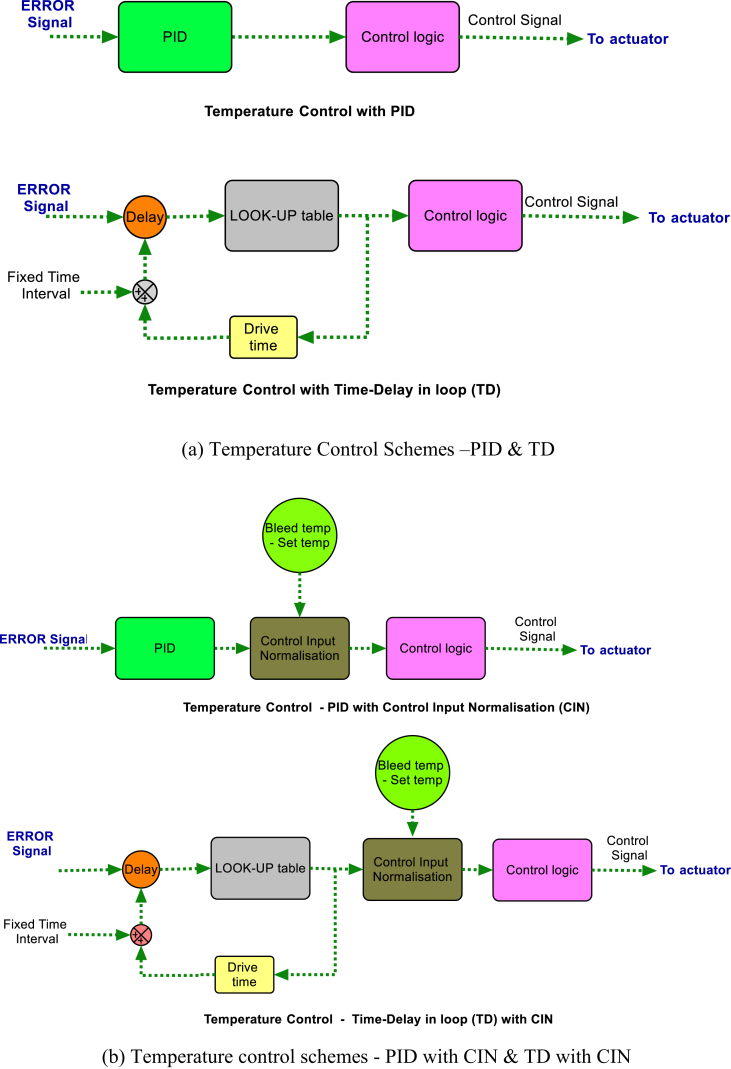
Temperature control schemes.

Broadly two categories of control scheme, conventional PID controller, and a novel TD (Time Delay) are deliberated and presented as shown in Figure 2a [18]. Both schemes use temperature error signal as input and send the control signal to the actuator. In the second method, the novel control strategy method, i.e. control with time delay, a finite time delay is introduced from the look-up table. Then, the corresponding control signal is generated. Thus control signal will drive the valve for a proportionate period as per the drive-time from the look-up table. The dwell or time-delay is kept constant for the entire analysis of the control system. The following control strategies are considered:

a. PID Control

Here, control drive signal is calculated based on PID parameters. The gain values used are 1.0, .01, and 0.1 for P, I, and D respectively. Based on the error, the control signal is calculated, which is continuous, and provided to the controller. Then, the controller sends the valve drive angle and the direction of valve movement and accordingly directs the TCV [22, 23].

b. Time-delay in loop control

This Time-Delay (TD) control is a novel method where a fixed time delay is injected between consecutive control signals. Other processes are same as that of PID method. Unlike PID, the actuation of TCV is not continuous [9, 10]. From a look-up table, the controller gets the control signal drive time for the calculated error value. The drive time is then further added with the fixed time period or delay to obtain the cumulative time period or delay. This total time period or time-delay is considered as the time gap between the consecutive processing of input data.

c. Time-delay with CIN

In this control, control signal is normalised by taking the input variation. The difference between control input parameter, and the set point of control signal was taken and then multiplied with controller output to generate compensation signal [18]. The schematic of this CIN method is given in Figure 2(b).

d. PID control with CIN

Since PID is a continuous control, the controller takes the error and directly commands the TCV based on PID output. But, here, the control input compensation signal was used along with PID control.

e. Variable TD

This is an improved version of the Time-delay control. In this, the time delay or dwell is not constant and varies with respect to input variations. A separate look-up table is created to provide the variable time delay based on input parameter and its rate of change. The purpose of having variable time delay is to avoid unnecessary dwelling of the control actuator.

f. Variable TD with CIN

As similar to TD with CIN, in this control, CIN is added into this variable time delay control. Linear Quadratic Regulator (LQR) is not considered for this study as it is useful for linear systems and not effective to control complex or nonlinear structures. Also, the correction of Q and R matrices is inevitable and requires huge amount of data to handle them and the model based LQR works on the basis of state space system which is not always possible [13].

## 4 System model

**Figure 3:**
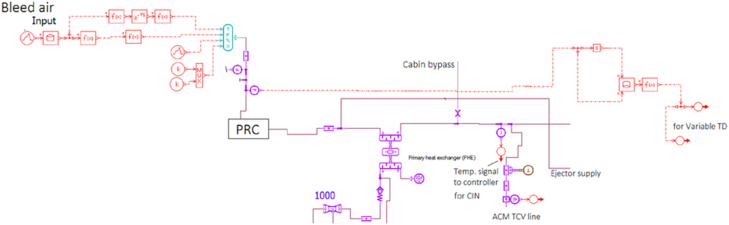
System model revision in AMESim.

In order to simulate and verify the temperature control strategies, the model chosen got updated, from previous work [18, 24, [30], [31]], in temperature control circuit to include novel control strategies viz. Control Input Normalisation (CIN) and Variable TD. As engine rpm rises, the PHE outlet temperature also rises and subsequently increases the control signal error of the TCS. To avoid sudden change in the TCV drive signal due to this error, control signal is compensated by bleed temperature measured at the PHE outlet. So, in CIN, the PHE outlet temperature was used to compensate the control signal. This compensated control signal is inversely proportional to the PHE outlet temperature. A part of updated model is provided in Figure 3. The input to the model was the bleed air and the output was the cabin inlet temperature. One dimensional flow was assumed. The library components, already available were used with minor modifications. Bleed pressure was measured at the upstream of PRC and used along with its rate of change as input to generate variable time delay. This variable TD signal was then given to the controller.

Electrically actuated butterfly valve was chosen as TCV and its drive is actuated by continuous signal from the controller in time step intervals according to the temperature error i.e. time steps is derived from temperature error, which is fed into a look-up table. By varying the time steps or drive time, in milliseconds, the actuator rotates in either open or close direction based on open/close drive signal. This effective area is then a function of valve rotation angle, *θ* i.e. how much the valve is opened; *A*_*o*_ is the maximum cross sectional area of the valve, and is provided in Eq. (2).

(2) $$A_{eff}=A_{0}\;\left(1-\cos\;\theta \right)$$

The mass flow through this valve depends mainly on effective flow area, upstream air pressure and temperature. The mass flow through this valve is given by Eq. (3).

(3) $$w_{v}=\frac{A_{eff}p_{u}}{\sqrt{T_{u}}}{\left[\frac{2\gamma g}{\left(\gamma -1\right)R}\left(\phi^{2/\gamma }-\phi^{\left(1+\gamma \right)/\gamma }\right)\right]}^{\frac{1}{2}}$$

where, $\phi =\frac{p_{dn}}{p_{u}}$;

$A_{eff}–effective\;flow\;area$;
R – gas constant;
γ−Specificheatratio;
$p_{u}$ – downstream pressure
$p_{u}\;and{\;T}_{u}\;are\;upstream\;pressure\;and\;temperatures\text{.}$

## 5 Methodology

**Figure 4:**
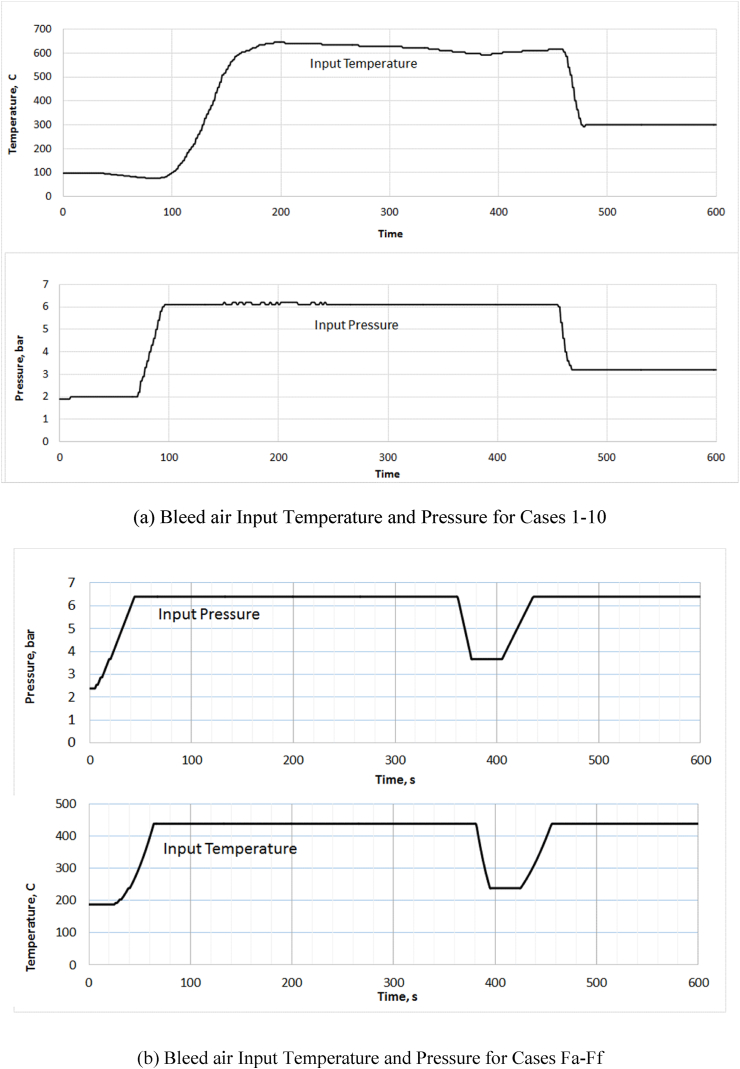
Bleed profile.

For model validation, the system was first simulated for on ground conditions and validated with actual data. The input bleed conditions are given in Figure 4(a) for cases 1 to 10 and Figure 4(b) for cases Fa to Ff. Then the model was first simulated in TD & PID controls with various time delay and having two different valve drive timings as given in Table 1 below. Cases 1 to 8 were simulated based on novel time-delay control strategy while cases 9 & 10 were simulated based on PID control. Also, six separate cases were simulated for in-air operations with different temperature control strategies i.e. PID, TD, PID with CIN, TD with CIN and variable TD, variable TD and variable TD with CIN. These simulation cases are summarised in Table 2. For the simulation of these cases, steady level flight at 3000 m ASL, 0.701 bar A ambient pressure and 0.930 bar A of cabin pressure were considered while the speed of the aircraft was assumed as 300 kmph Calibrated Air Speed (CAS). The initial temperature and the ambient temperature were considered as 10 °C and the specific humidity at the inlet was assumed as 1.9 g/kg of dry air.

**Table 1:** Cases for TD and PID comparison.

Parameters	Case 1	Case 2	Case 3	Case 4	Case 5	Case 6	Case 7	case 8	Case 9	Case 10
Delay period in sec	1	2	4	6	1	2	4	6	0 (PID)	0 (PID)
Valve drive speed in sec	10	10	10	10	6	6	6	6	10	6

**Table 2:** Cases for various control strategies.

Cases	Engine RPM %	Input Pressure in bar	Input Temperature in ^o^C	Temp. control
Case-Fa	75 to 97	2.8 to 6.4	175 to 440	TD (4 s)
Case-Fb	75 to 97	2.8 to 6.4	175 to 440	PID
Case-Fc	75 to 97	2.8 to 6.4	175 to 440	TD with CIN
Case-Fd	75 to 97	2.8 to 6.4	175 to 440	PID with CIN
Case-Fe	75 to 97	2.8 to 6.4	175 to 440	Variable TD
Case-Ff	75 to 97	2.8 to 6.4	175 to 440	Variable TD with CIN

The gain values of PID controller were first estimated based on Ziegler-Nichols method [23]. A sampling rate of four samples per sec is considered by taking into account the practical nature of measuring temperature and pressure on aircraft. So, the time scale in the simulation is four times of the actual time. The model simulation period of 600 s is equal to 150 s of actual time. A separate look-up table, Table 3, is used to provide the variable time delay based on input parameter and its rate of change.

**Table 3:** Look-up table for variable TD.

		Input Pressure in bar A						
		2.6	3.8	5	6.2	7	7.1	7.2
Rate of change of input pressure	-3	1.00	0.71	0.45	0.33	0.28	0.25	0.25
	-2	2.00	1.67	1.00	0.63	0.42	0.31	0.25
	-1	4.00	4.00	2.00	1.00	0.50	0.33	0.25
	0	4.00	4.00	2.00	1.00	0.50	0.33	0.25
	1	4.00	4.00	2.00	1.00	0.50	0.33	0.25
	2	2.00	1.67	1.00	0.63	0.42	0.31	0.25
	3	1.00	0.71	0.45	0.33	0.28	0.25	0.25

## 6 Results and discussion

The 10 cases, as given in Table 1, are simulated and the results are compared for the turbine outlet temperature and TCV positions. It shows that longer the delay time, the slower the response and produces wavy temperature profile. On the other hand, shorter delay time gives quicker response but, it increases the actuator total movement and subsequent energy consumption.

**Figure 5:**
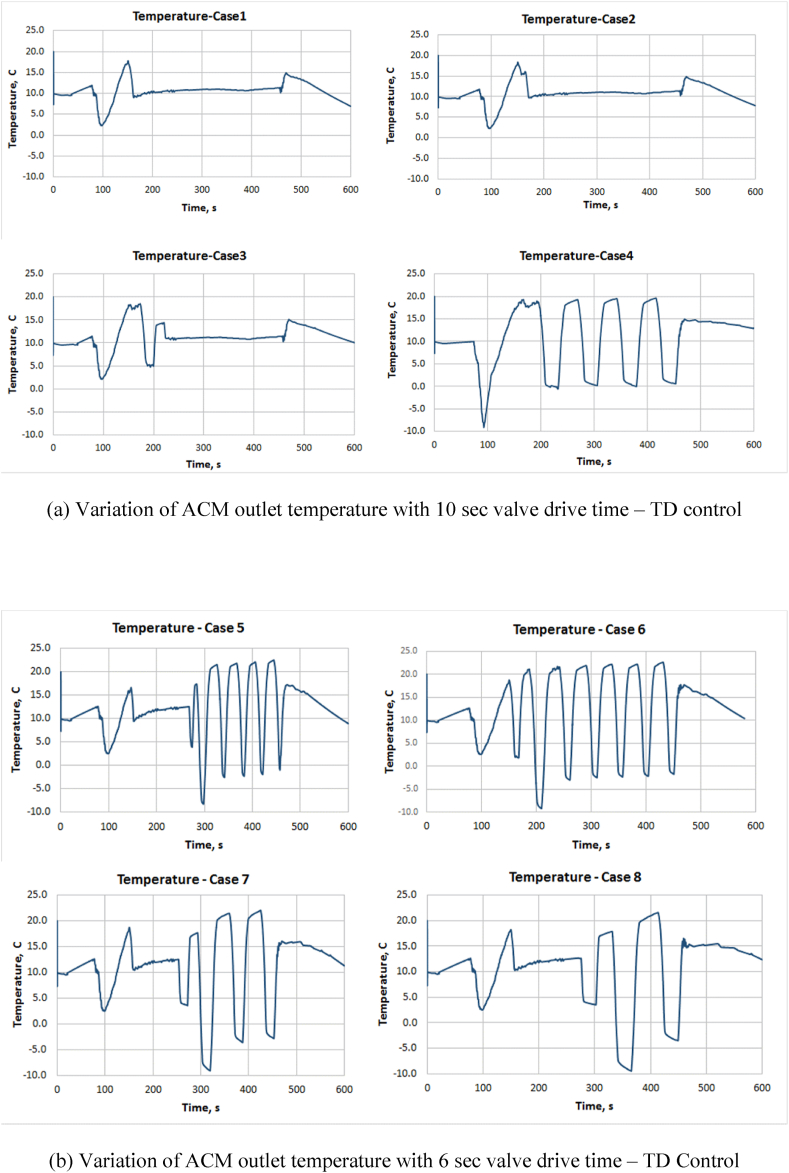
Variation of ACM outlet temperature – TD control.

**Figure 6:**
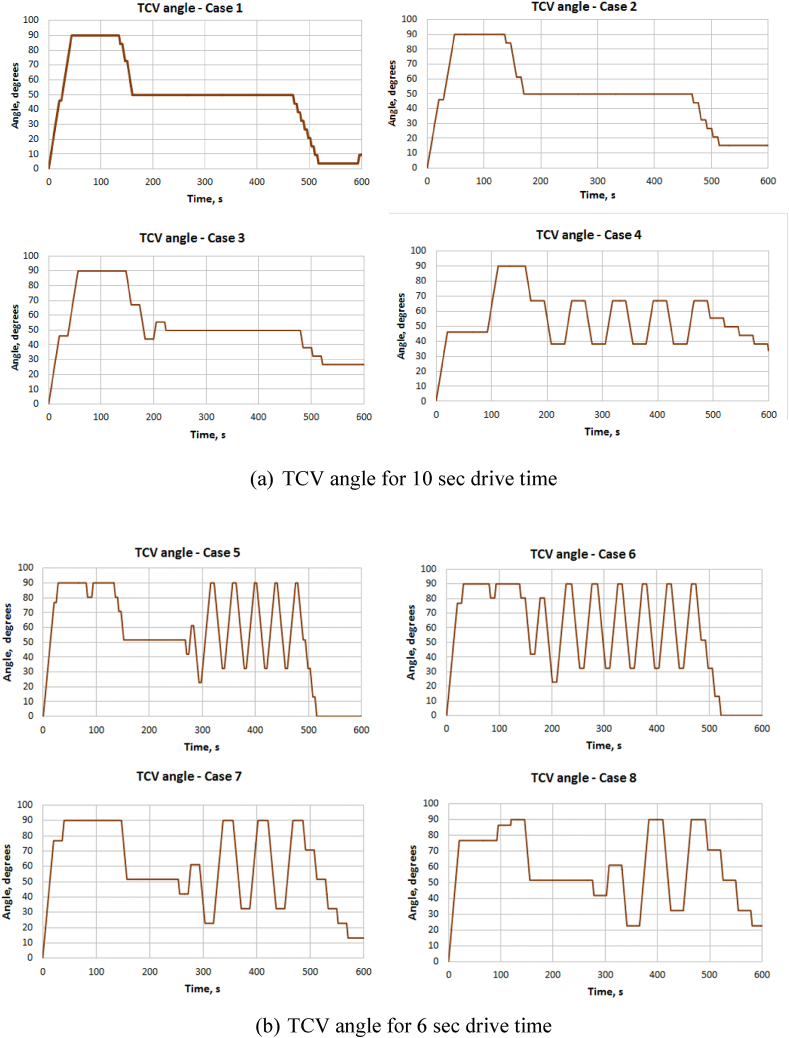
Variation of TCV angle for (a) 10 s and (b) 6 s drive time– TD control.

**Figure 7:**
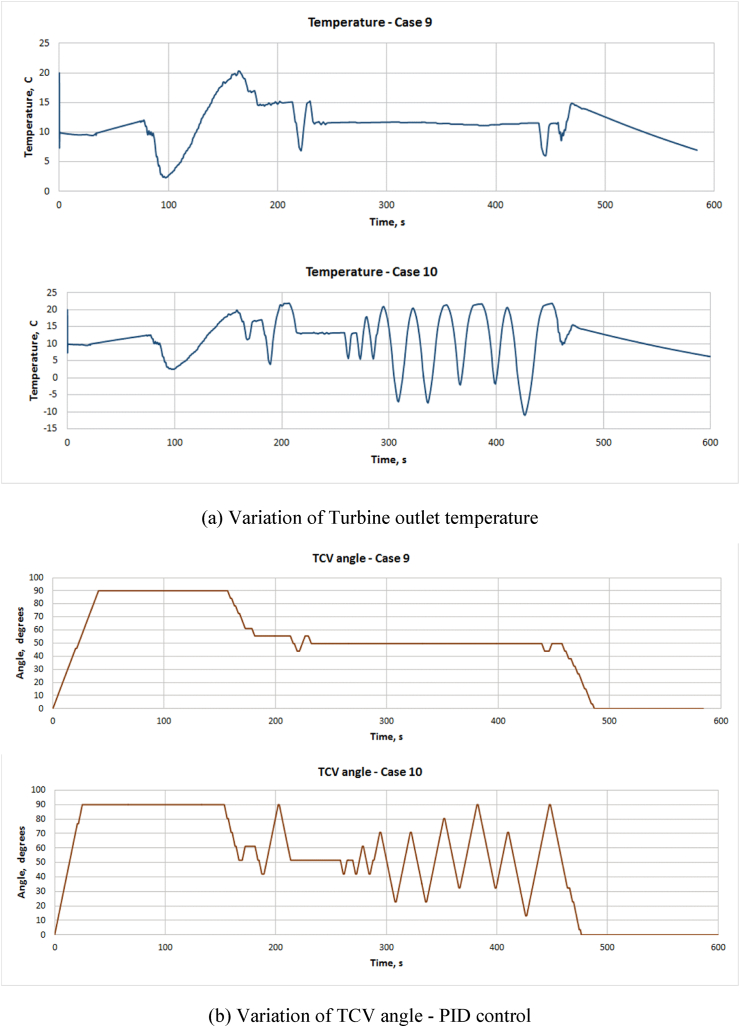
Variation of (a) ACM outlet temperature and (b) TCV angle for PID control with 10 s (case 9) & 6 s (case 10) valve drive time.

**Figure 8:**
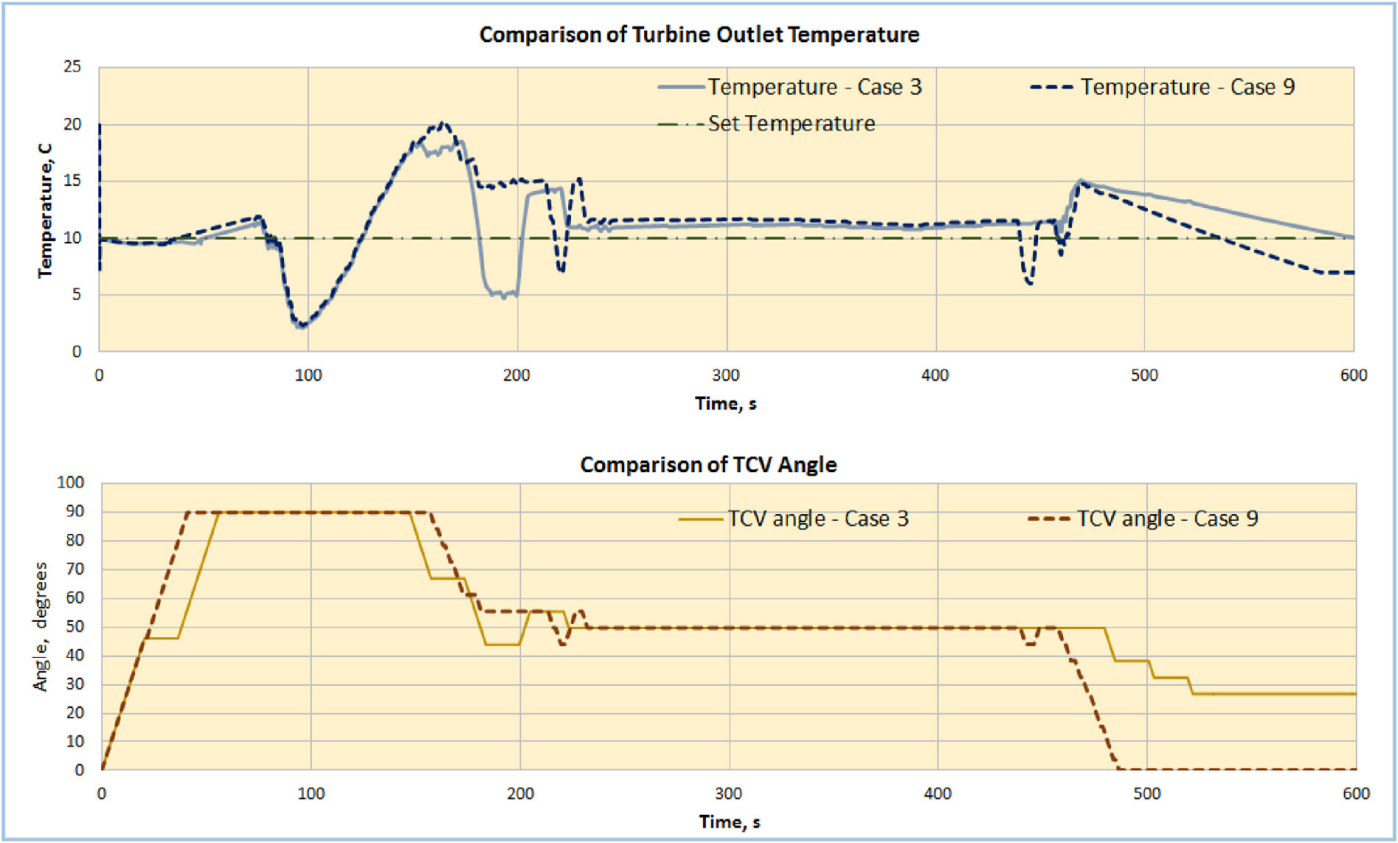
Comparison of ACM outlet temperature and TCV angle for case 3 & 9.

Figures 5, 6, 7 and 8 show the temperature variation at ACM outlet temperature and TCV position for all cases of TD and PID control strategies. Figure 5(a) and (b) shows the variation of ACM outlet temperature for cases 1 to 8 and Figure 6(a) and (b) shows variation of TCV angle for cases 1 to 8. Figure 7(a) shows ACM outlet temperature and Figure 7(b) shows TCV angle for cases 9 &10 respectively. From the results, it is clear that PID method is quicker as found in case 10 in comparison with TD control. PID has large overshoot while TD control has smaller overshoot. Also, the settling time is more for PID control. Also, in comparison with TD method, PID control method has more duration of the TCV movement. This will contribute to continuous operation of the valve and more energy consumption in PID control. Due to this, TCV and its driving mechanism will get mechanical wear and tear which further lead to failure of the components and the actuator.

Even in TD control method, the temperature fluctuation is inevitable and the same is extensively observed in cases 4 to 8 due to the combination of shorter drive time of the valve and the shorter dwell period. These are not observed with cases 1 to 3. Cases 1 to 3 have almost same end limits but case 3 has better performance at the end part of the simulation than others.

Also, the temperature fluctuation in case 9 has sharp ups and downs than in case 3. TCV also reaches end limits frequently than case 3. In case 10, PID control has drastic and sharp temperature fluctuations. On the whole, cases 1, 2, 3 and 9 seem to be better than other cases. But, considering the practical aspects of TCV, the case 3 is seem to be better than others.

Figure 8 shows the comparison of ACM outlet temperature and TCV angle for both methods of control. It shows that though the profiles apparently match between two methods, PID method lags in controlling the temperature during the end portion of simulation. But, TD control, i.e. case 3, keeps the temperature below the limit and has smoother profile.

### 6.1 Control input normalisation (CIN)

As PID control doesn’t maintain the temperature within the limit and doesn’t have smoother profile, TD control is the better option for cabin temperature control of a fighter aircraft. Nevertheless, it has slight temperature oscillations, and undesirable TCV rotation.

**Figure 9:**
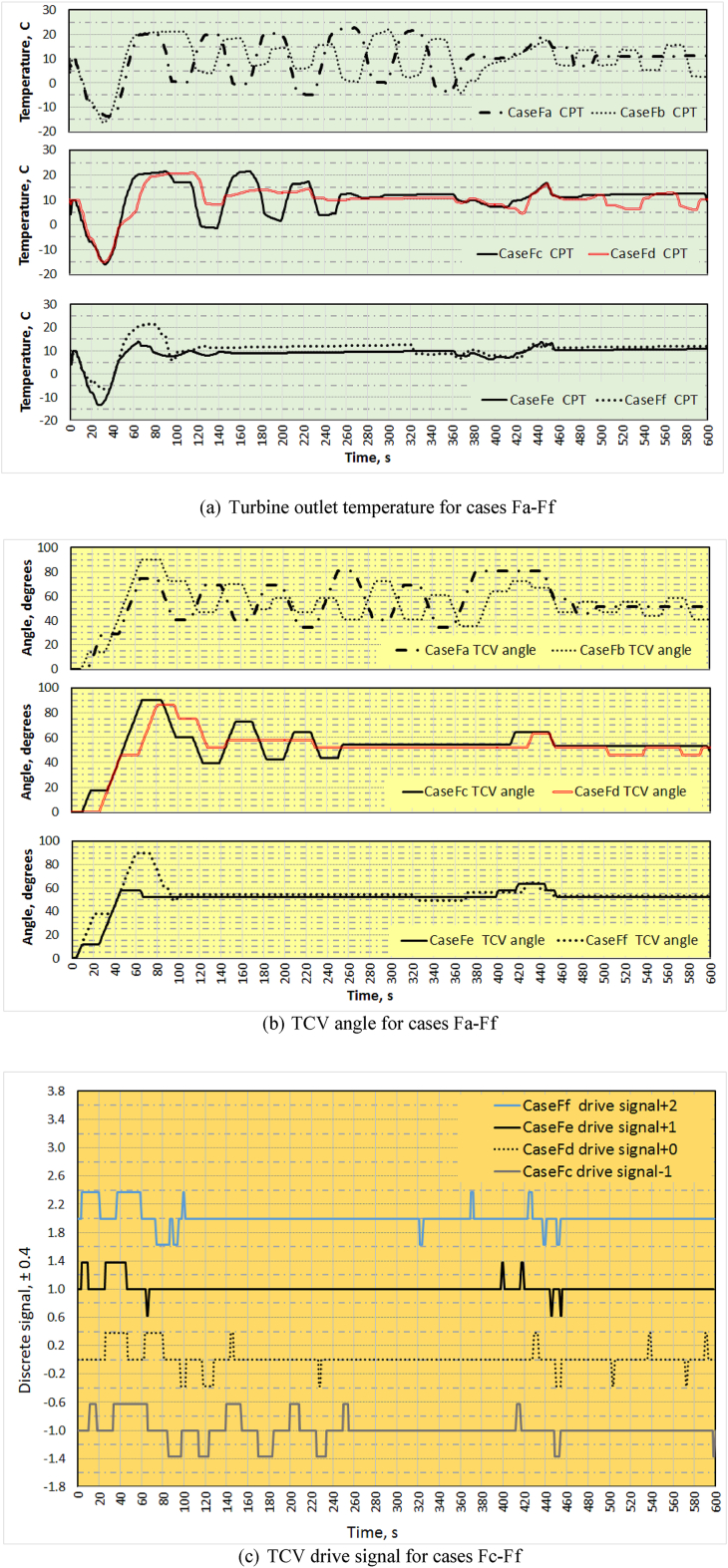
Comparison of (a) Turbine outlet temperature, (b) TCV angle and (c) TCV drive signal for cases Fa-Ff - simulated.

So, to upgrade the control, a control input compensated variant, Control Input Normalisation (CIN) signal, was introduced. This compensated signal is derived based on the temperature difference PHE outlet and the required ACM outlet. This signal is then multiplied with controller output to drive the TCV. The results of the simulation for all flight cases, Fa to Ff, are presented in Figure 9. The variation of turbine outlet temperature, TCV angle and TCV drive signal are shown in Figure 9(a), 9(b) and 9(c) respectively. As the variation in TCV drive signal is huge in cases Fa & Fb, the same is omitted in Figure 9(c). It is found that TD with CIN maintains temperature at the ACM outlet with less oscillation and less TCV movement and produced good results in comparison with other methods. The performance comparison is given in Table 4. Although this method go beyond in overshoot in comparison to TD without CIN and has good performance and stability index.

**Table 4:** Performance comparison.

Parameters	PID Control	Time-delay Control	PID with CIN	Time-delay with CIN
Performance index	0.56	0.82	0.66	0.90
Overshoot	1.1	0.8	1.0	1.0
Stability index	0.1	0.86	0.14	0.90
No of valve actuations	310.8	81.5	271	71

### 6.2 Variable time-delay

To improve the Time-delay method further, the time-delay considered was not kept constant and varied based on the input parameter. The bleed pressure and its rate of change were considered as the driving forces to determine the time-delay. A look-up table, Table 3, was then created to get directly the variable time-delay for a specific bleed pressure and its rate of change at a particular instant. The results of the simulation for all flight cases with variable TD are presented in Figure 9 with cases of Fe &Ff. It is found that though PID with CIN gives good result initially up to 360 s, but due to variation in bleed input, this doesn’t control the temperature fluctuations better than variable TD. Also, based on analysis of the number of times TCV was driven, it was observed that variable TD control gives better result than variable TD along with CIN. The comparison of the performance is given in Table 5.

**Table 5:** Performance comparison – variable TD.

Parameters	Variable Time-delay	Variable Time-delay with CIN
Performance index	0.95	0.89
Overshoot	0.67	1.0
Stability index	0.88	0.82
No of valve actuations	70	100

**Figure 10:**
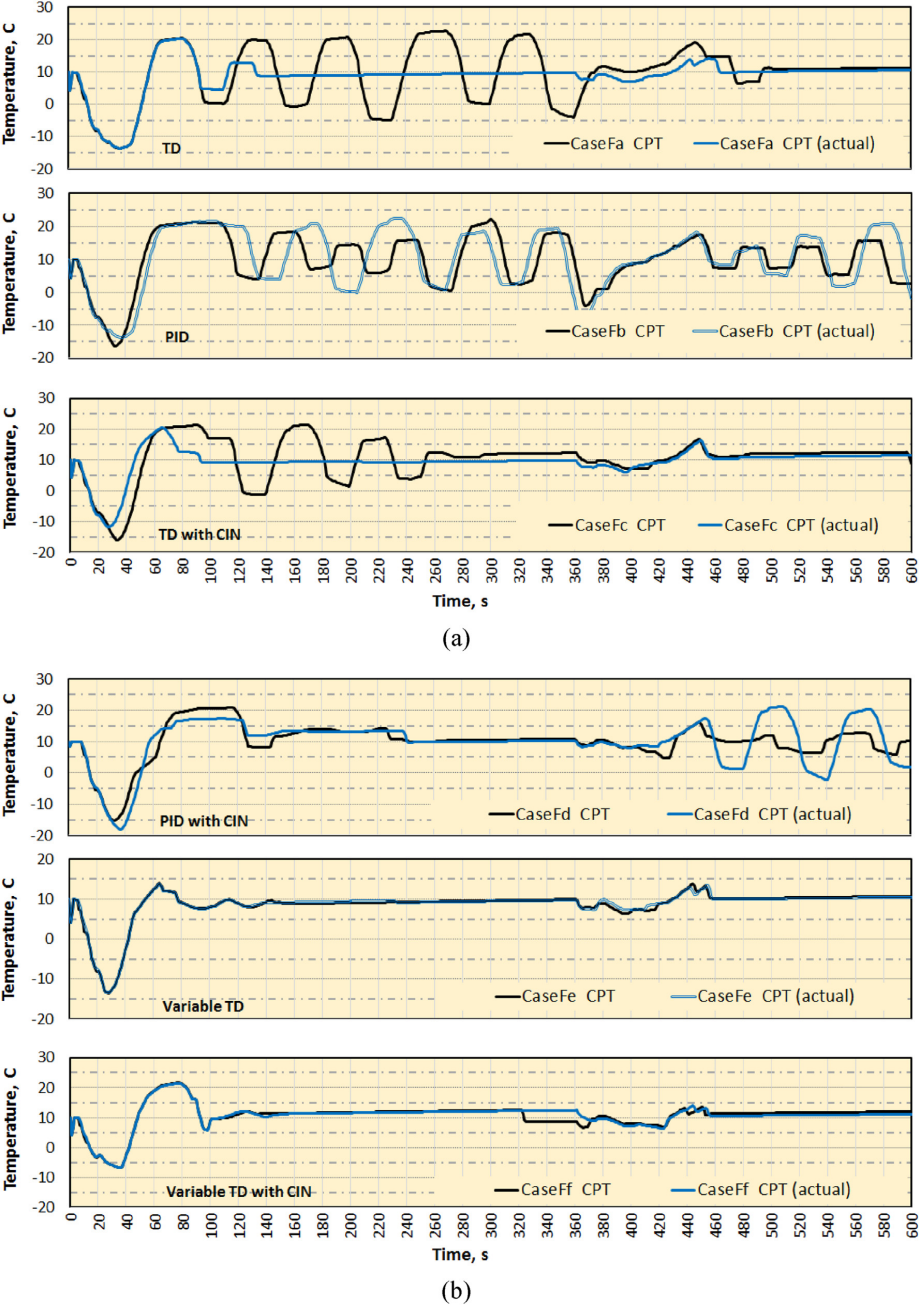
Comparison of Turbine outlet temperature for cases (a) Fa-Fc and (b) Fd-Ff with actual data.

On the whole, TD control has good control characteristics than PID and in particular, variable TD control provides better cabin temperature control over other methods. Though input compensated control gives comparatively better results than mere either PID or TD control, with the use of variable TD control, both variable TD and variable TD with CIN the TCS produce better results. The same is verified with the help of actual data in comparison with simulated data. The comparison of turbine outlet temperature is made between simulated and actual data for cases Fa to Fc as shown in Figure 10a and for cases Fd to Ff in Figure 10b. As the measurement of actual drive signal for the TCV is not possible, the same is not included for the comparison. However, comparing minor overshoots and number of valve actuations, variable TD appears to be better than variable TD with CIN.

## 7 Conclusion

Transient characteristics of a combat aircraft ECS are studied by 1D simulation. The temperature control methods to maintain required cabin temperature are simulated broadly for four schemes. The complexities involved in the design of cockpit supply air temperature control system are realised during the development of the model. The simulated results of different control methods comparing the ACM outlet temperature and TCV angle are provided. Result shows that TD control provide better results than other methods. Case 3 and 9 give better comparison of the same, i.e. comparing the time-delay control and PID control. It is found that though the profiles apparently match between two methods, the number of valve drive signal is more in case 9 than case 3, and case 9 doesn’t maintain the temperature at the end. In addition to the above, an improved version of TD control, a novel method, variable time-delay control has been evaluated and produced better results than all other methods. By comparison of all control strategies aforementioned it is proposed that variable TD is the better option than other methods. This method of control improves the performance of the ECS and reduces the chances of failure of control components in ECS. The model thus developed can further be used to quickly generate results for validation studies of new concepts.

## Declarations

### Author contribution statement

Sathiyaseelan A: Performed the experiments; Analyzed and interpreted the data; Contributed reagents, materials, analysis tools or data.

Arul Mozhi Selvan V: Conceived and designed the experiments; Wrote the paper.

### Funding statement

This research did not receive any specific grant from funding agencies in the public, commercial, or not-for-profit sectors.

### Data availability statement

The data that has been used is confidential.

### Declaration of interest’s statement

The authors declare no conflict of interest.

### Additional information

No additional information is available for this paper.
